# Supplementary material for: Structural insights into mechanism and specificity of the plant protein O-fucosyltransferase SPINDLY
Source: Nat Commun. 2022 Dec 2;13:7424. doi: 10.1038/s41467-022-35234-0 (PMC9715652; doi:10.1038/s41467-022-35234-0)
Supplement: Supplementary file 1 — Supplementary Information [file 41467_2022_35234_MOESM1_ESM.pdf]

Supplementary Information

Structural insights into mechanism and specificity of the plant protein O-fucosyltransferase SPINDLY

Li Zhu, Xiting Wei *et al*

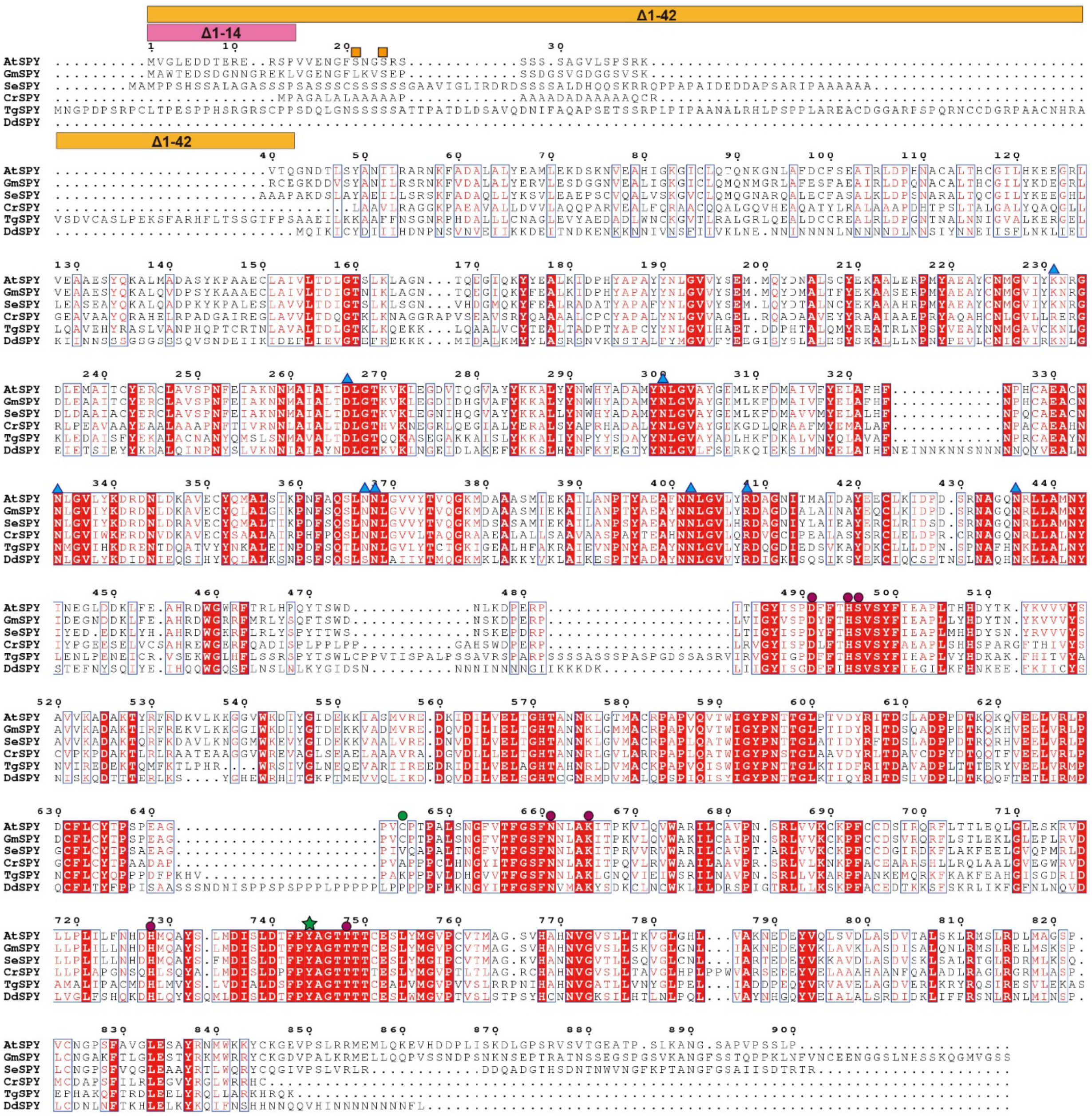

Supplementary Fig. 1. Sequence alignment of SPY proteins from plant (*Arabidopsis*, *Glycine max*), bacteria (*Synechococcus elongatus*), algae (*Chlamydomonas reinhardtii*), and protist (*Toxoplasma gondii*, *Dictyostelium discoideum*). The orange squares, blue triangles and red dots indicate target residues for self-fucosylation, residues involved in protein substrate binding, residues involved in glycan donor substrate binding and catalysis in *Arabidopsis* SPY, respectively. The green dot and green star indicate C645 and Y744 in *Arabidopsis* SPY, respectively.

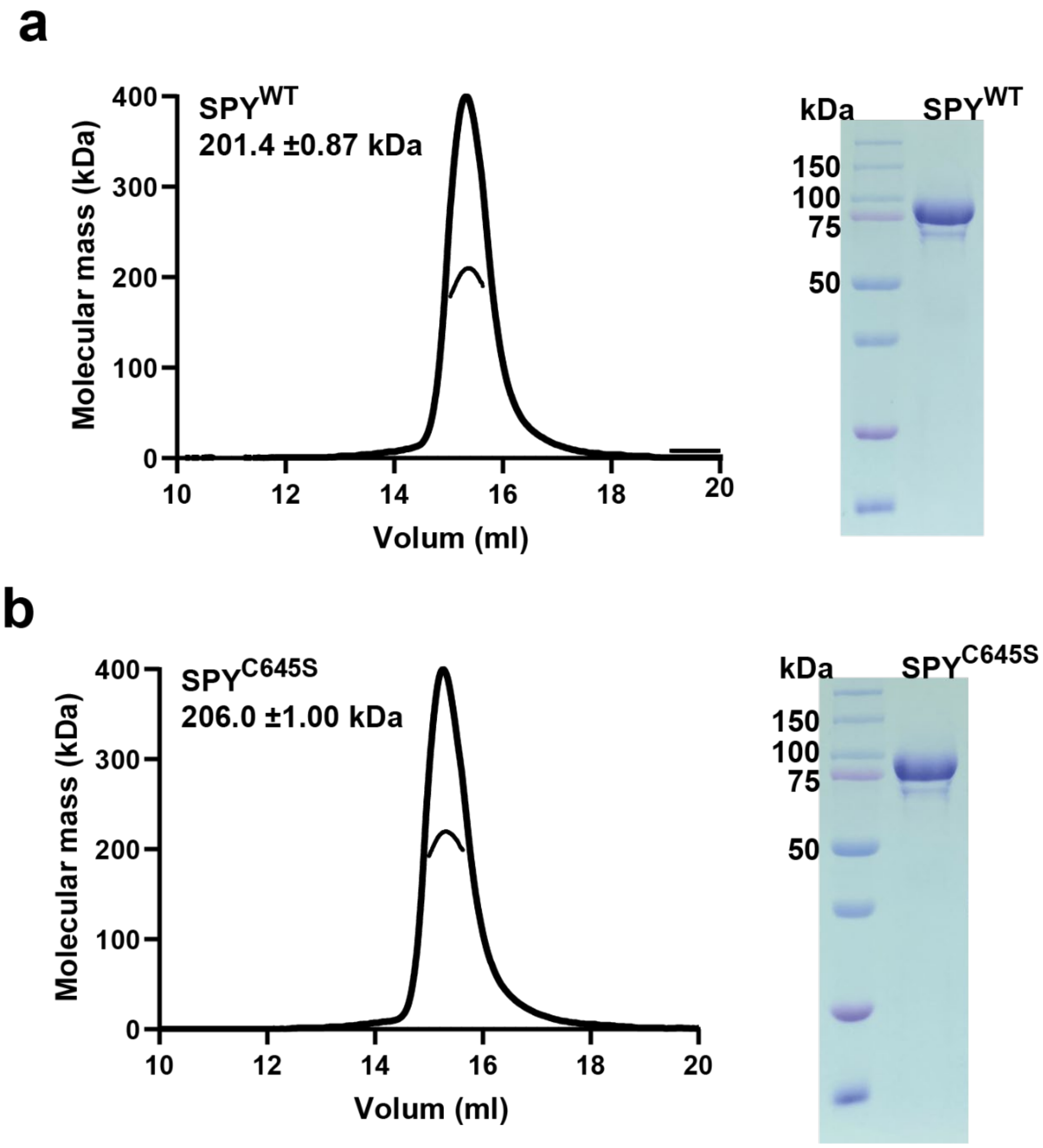

Supplementary Fig. 2. SEC-Mals characterization. a, The measured molecular weight of wild type Arabidopsis SPY (SPY^{WT}) in solution is around 201 kDa, that is about twice of the SPY monomer. It indicates that SPY^{WT} is a dimer. Experiments were independently repeated three times with similar results. **b,** The measured molecular weight of SPY^{C645S} in solution is about 206 kDa, indicating it also adopts dimeric form. Experiments were independently repeated three times with similar results.

a

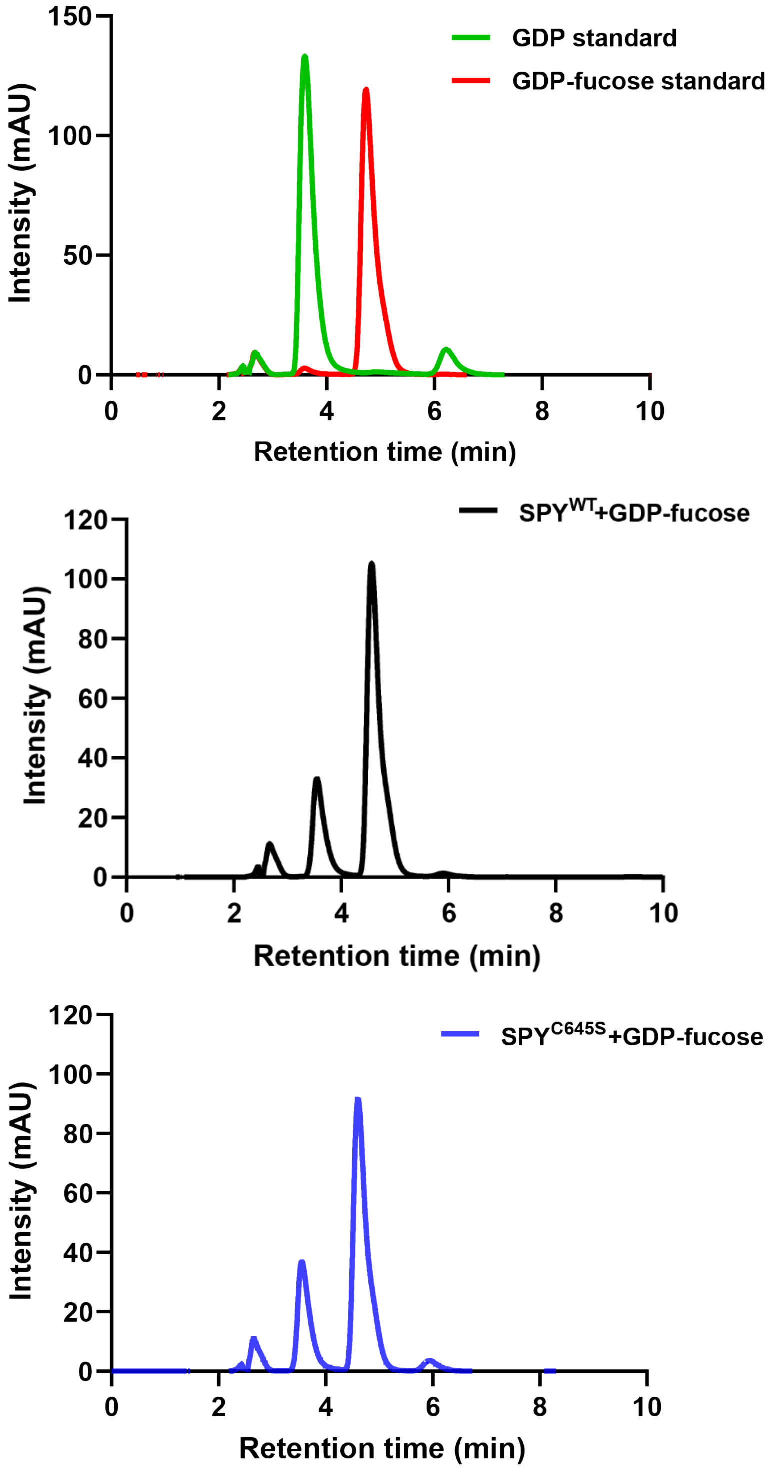

GDP standard
GDP-fucose standard
Intensity (mAU)
Retention time (min)
SPY^WT+GDP-fucose
Intensity (mAU)
Retention time (min)
SPY^C645S+GDP-fucose
Intensity (mAU)
Retention time (min)

b

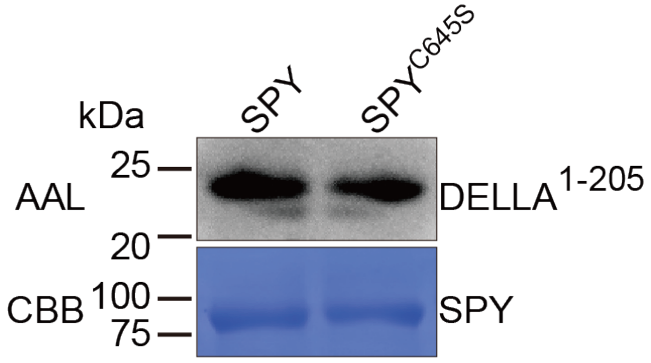

Supplementary Fig. 3. *In vitro* enzyme activity of *Arabidopsis* SPY. a, *In vitro* SPY activity assay by HPLC. The standard GDP and GDP-fucose were used as references (upper panel). Both the wild type SPY (SPY^{WT}, middle panel) and SPY^{C645S} (lower panel) exhibit enzyme activity in presence of GDP-fucose. Experiments were independently repeated three times with similar results. **b,** *In vitro* fucosylation activity assay by Western blotting with biotinylated AAL showed that SPY^{WT} and SPY^{C645S} possess similar glycosylation activity toward $DELLA^{1\text{-}250}$. Upper panel shows western blotting with biotinylated AAL. Lower panel shows Coomassie blue staining. Experiments were independently repeated three times with similar results. Uncropped images of Western blotting membranes and gels are available as source data.

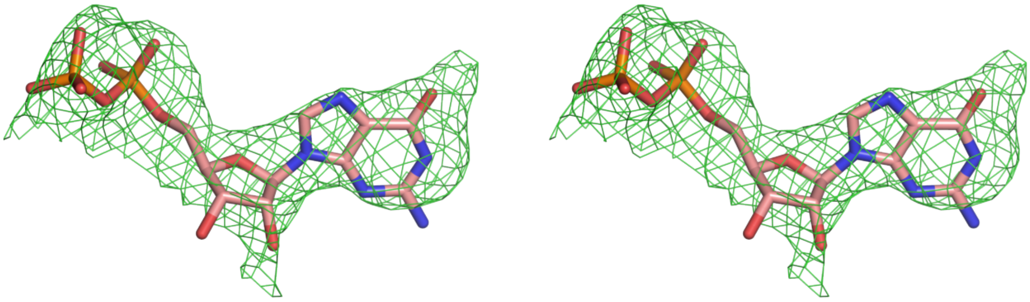

Supplementary Fig. 4. Stereo representation of the electron density map of GDP bound in *Arabidopsis* SPY. The *Fo-Fc* electron density (2.0 σ level) of GDP is shown in green.

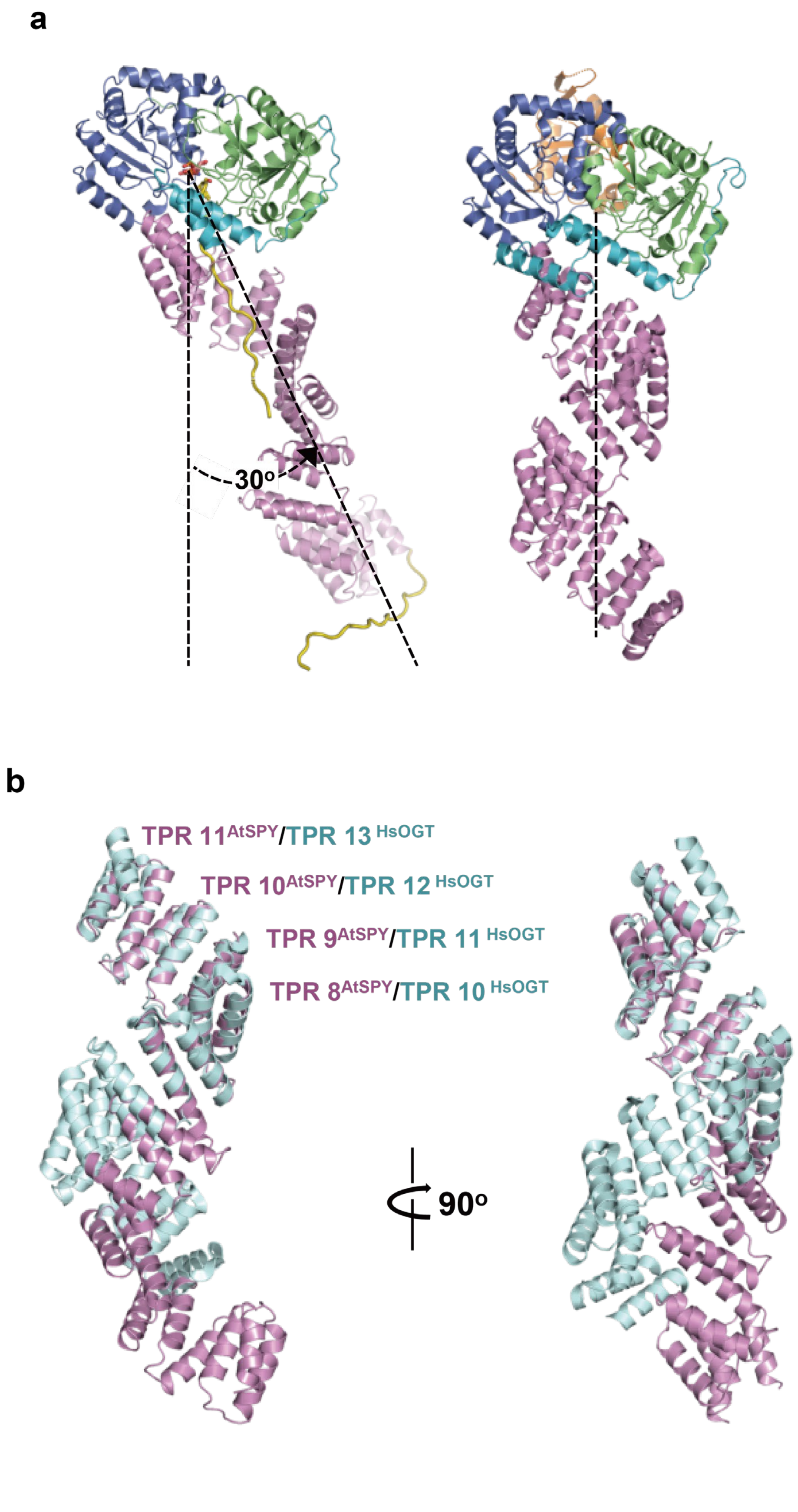
a
30o
b
TPR 11AtSPY/TPR 13 HsOGT
TPR 10AtSPY/TPR 12 HsOGT
TPR 9AtSPY/TPR 11 HsOGT
TPR 8AtSPY/TPR 10 HsOGT
90o

c

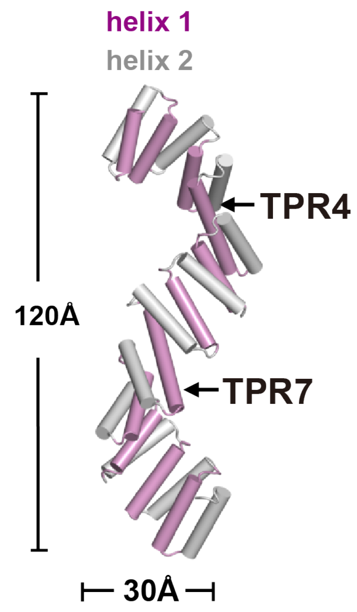

helix 1
helix 2
TPR4
TPR7
120Å
30Å

d

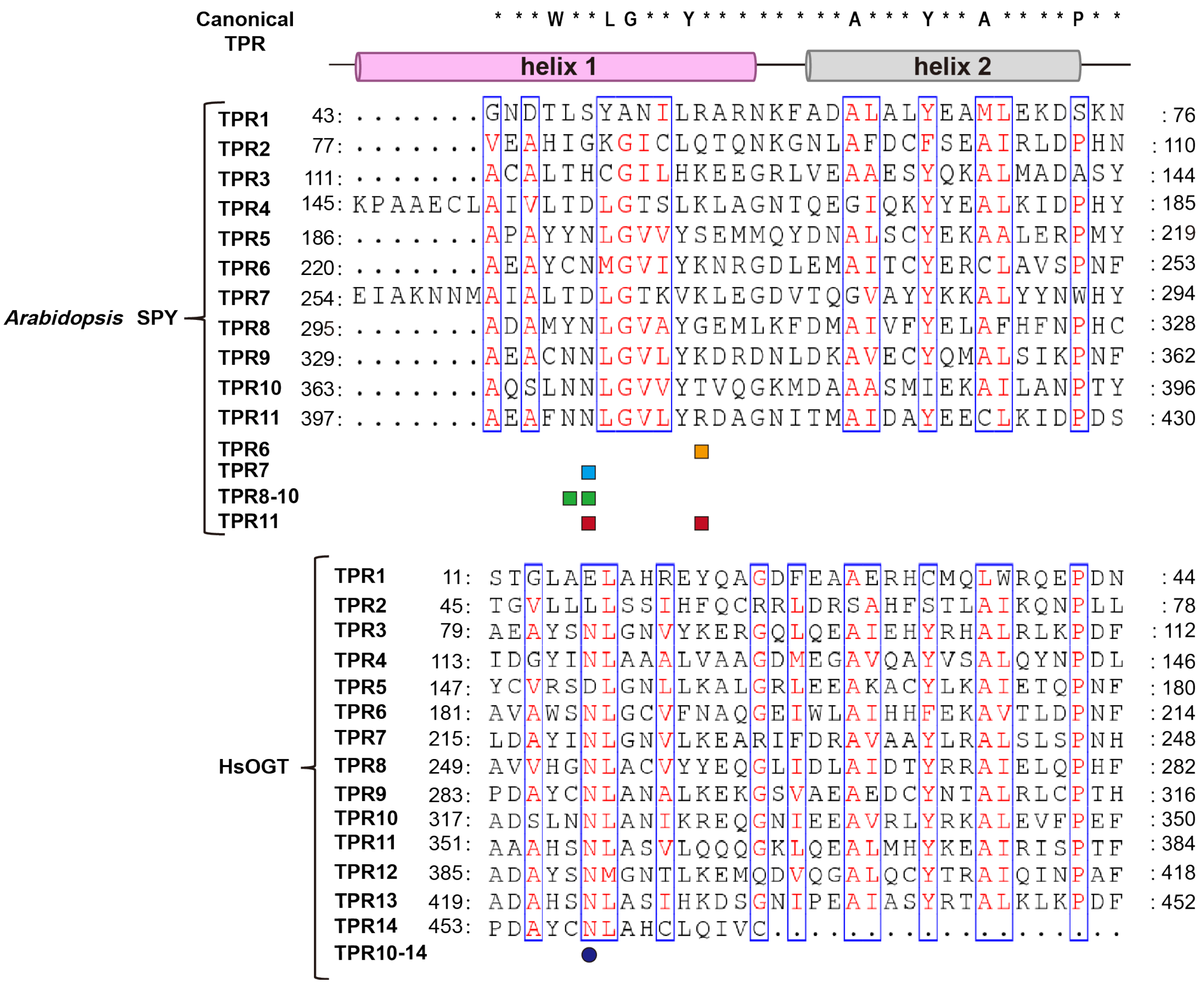

Canonical TPR
* * * W * * L G * * Y * * * * * * * * A * * * Y * * A * * * * P * *
helix 1
helix 2
Arabidopsis SPY
TPR1 43: ........GNDTLSYANILRARNKFADALALYEAMLEKDSKN : 76
TPR2 77: ........VEAHIGKGICLQTQNKGNLAFDCFSEAIRLDPHN : 110
TPR3 111: ........ACALTHCGILHKEEGRLVEAAESYQKALMADASY : 144
TPR4 145: KPAAECLAIVLTDLGTSLKLAGNTQEGIQKYYEALKIDPHY : 185
TPR5 186: ........APAYYNLGVVYSEMMQYDNALSCYEKAALERPMY : 219
TPR6 220: ........AEAYCNMGVIYKNRGDLEMAITCYERCLAVSPNF : 253
TPR7 254: EIAKNNMAIALTDLGTKVKLEGDVTQGVAYYKKALYYNWHY : 294
TPR8 295: ........ADAMYNLGVAYGEMLKFDMAIVFYELAFHFNPHC : 328
TPR9 329: ........AEACNNLGVLYKDRDNLDKAVECYQMALSIKPNF : 362
TPR10 363: ........AQSLNNLGVVYTVQGKMDAAASMIEKAILANPTY : 396
TPR11 397: ........AEAFNNLGVLYRDAGNITMAIDAYEECLKIDPDS : 430
TPR6
TPR7
TPR8-10
TPR11
HsOGT
TPR1 11: STGLAELAHREYQAGDFEAAERHCMQLWRQEPDN : 44
TPR2 45: TGVLLLLSSIHFQCRRLDRSAHFSTLAIKQNPLL : 78
TPR3 79: AEAYSNLGNVYKERGQLQEAIEHYRHALRLKPDF : 112
TPR4 113: IDGYINLAAALVAAGDMEGAVQAYVSALQYNPDL : 146
TPR5 147: YCVRSDLGNLLKALGRLEEAKACYLKAIETQPNF : 180
TPR6 181: AVAWSNLGCVFNAQGEIWLAIHHFEKAVTLDPNF : 214
TPR7 215: LDAYINLGNVLKEARIFDRAVAAYLRALSLSPNH : 248
TPR8 249: AVVHGNLACVYYEQGLIDLAIDTYRRAIELQPHF : 282
TPR9 283: PDAYCNLANALKEKGSVAEAEDCYNTALRLCPTH : 316
TPR10 317: ADSLNNLANIKREQGNIEEAVRLYRKALEVFPEF : 350
TPR11 351: AAAHSNLASVLQQQGKLQEALMHYKEAIRISPTF : 384
TPR12 385: ADAYSNMGNTLKEMQDVQGALQCYTRAIQINPAF : 418
TPR13 419: ADAHSNLASIHKDSGNIPEAIASYRTALKLKPDF : 452
TPR14 453: PDAYCNLAHCLQIVC..................
TPR10-14

e

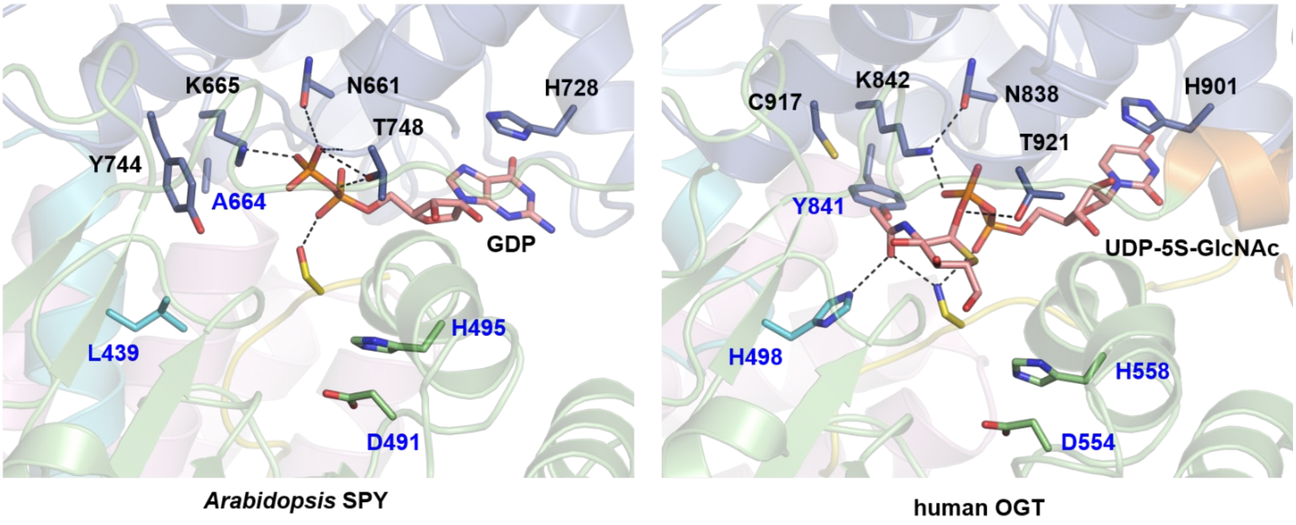

Supplementary Fig. 5. Structural comparison of *Arabidopsis* SPY and human OGT. **a,** Superposition of the *Arabidopsis* SPY (left panel) and human OGT[1] (PDB code 7NTF, right panel) structures was performed using the catalytic domains as references. The TPR domains are colored in pink, the connector regions are colored in cyan, the N-Cat lobes are colored in green, the C-Cat lobes are colored in blue. The N-terminal loop in Arabidopsis SPY is colored in yellow. The intervening domain in human OGT is colored in orange. **b,** Superposition of the TPR domains in *Arabidopsis* SPY (pink) and in human OGT (PDB code 7NTF, pale cyan). TPRs 8-11 in *Arabidopsis* SPY and TPRs 10-13 in human OGT are labeled. **c,** Cartoon representative of the TPR domain in *Arabidopsis* SPY. Helix 1 and helix 2 in each TPR unit are colored in pink and gray, respectively. TPR 4 and TPR7 are labeled. **d,** Structure-based sequence alignment of the 11 TPR units in *Arabidopsis* SPY and 13.5 TPR units in human OGT. The canonical TPR consensus sequence and the secondary structure elements are indicated at the top of the figure. The residues involved in substrate recognition are labeled with colored squares and dots at the bottom. **e,** Close-up view of the active sites in *Arabidopsis* SPY (left panel) and human OGT (right panel). The *Arabidopsis* 'catalytic SPY'/GDP/'substrate SPY' structure was superposed with the human OGT/UDP-5S-GlcNAc/aaTAB1tide structure[2] (PDB code 4AY6) using catalytic domains as references. The acceptor serine in aaTAB1tide is replaced with an aminoalanine. Hydrogen bonds are depicted by dashed lines.

a

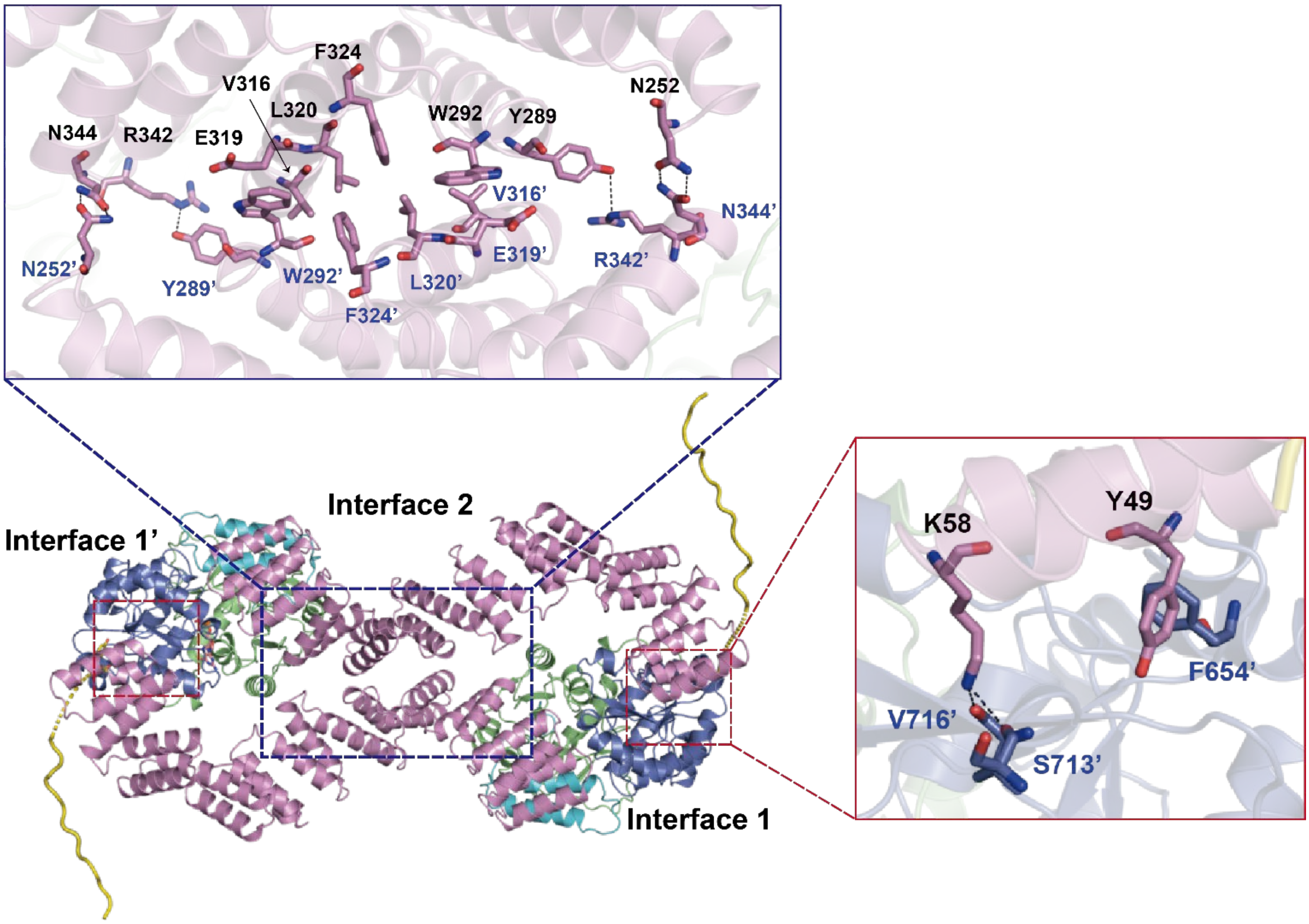

b

	Protomer 1			Protomer 2
Interface 1	TPR 1	K58	V716'	C-Cat'
		K58	S713'	
		Y49	F654'	
Interface 2	TPR 7	N252	N344'	TPR 9'
		Y289	R342'	
	TPR 8	W292	E319'	TPR 8'
		W292	V316'	
		L320	F324'	
		F324	L320'	
		V316	W292'	
		E319	W292'	
	TPR 9	R342	Y289'	TPR 7'
		N344	N252'	

c

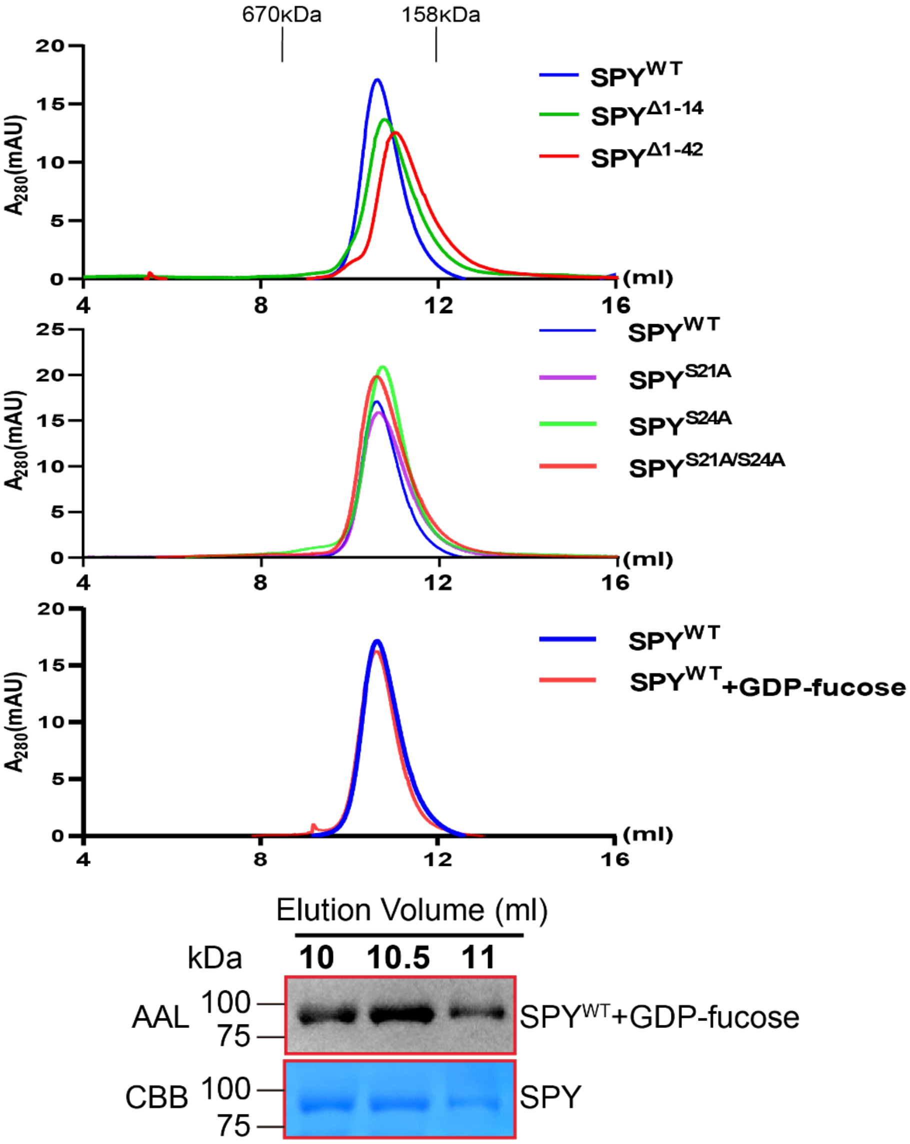

670κDa
158κDa
SPY^WT
SPY^Δ1-14
SPY^Δ1-42
A280(mAU)
(ml)
SPY^S21A
SPY^S24A
SPY^S21A/S24A
SPY^WT+GDP-fucose
Elution Volume (ml)
kDa
10
10.5
11
AAL
100
75
CBB
SPY

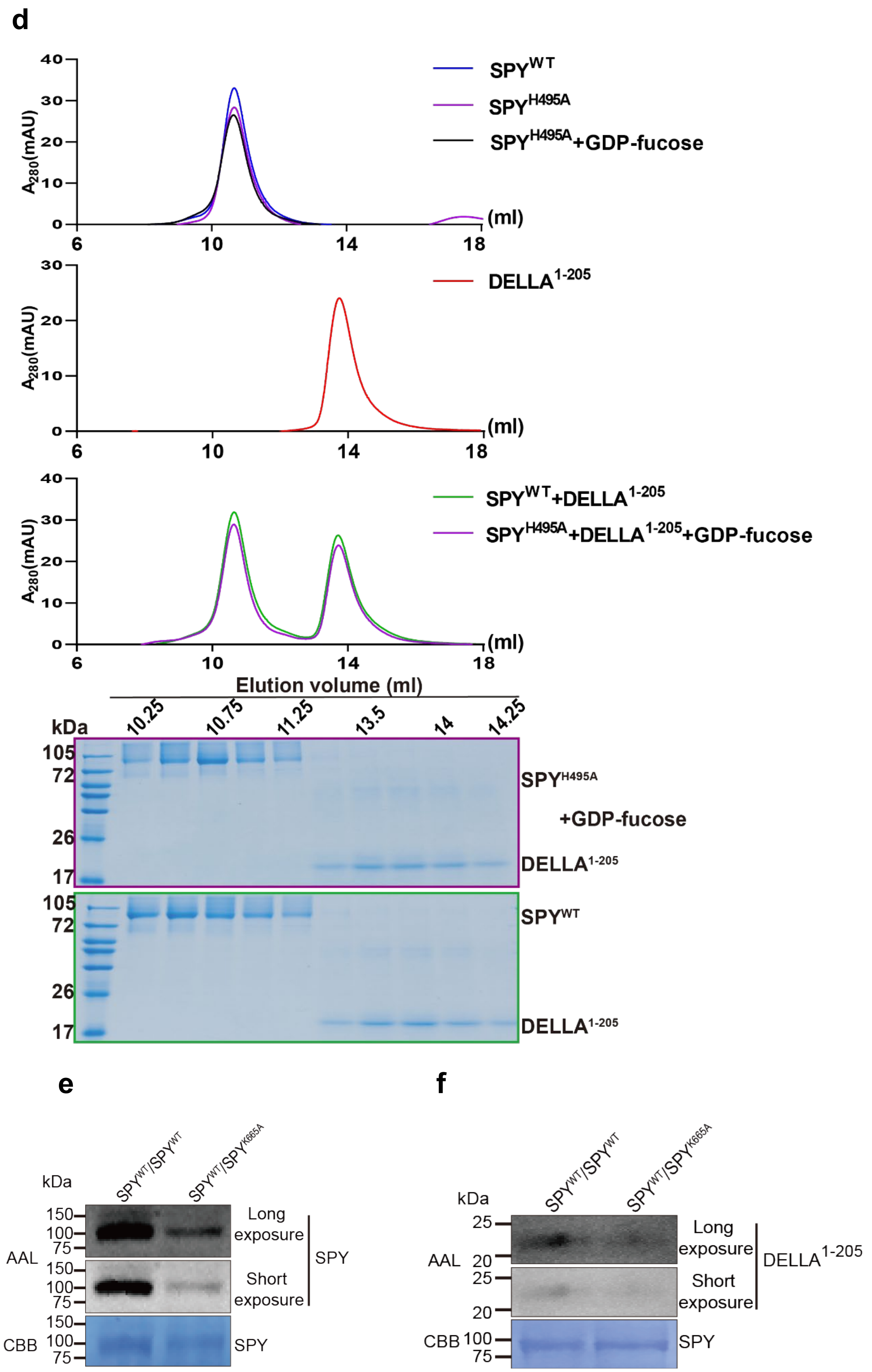

d
SPY^WT
SPY^H495A
SPY^H495A+GDP-fucose
A280(mAU)
(ml)
DELLA^1-205
SPY^WT+DELLA^1-205
SPY^H495A+DELLA^1-205+GDP-fucose
Elution volume (ml)
kDa
10.25
10.75
11.25
13.5
14
14.25
105
72
26
17
SPY^H495A
+GDP-fucose
DELLA^1-205
SPY^WT
DELLA^1-205
e
SPY^WT/SPY^WT
SPY^WT/SPY^K665A
kDa
150
100
75
Long exposure
Short exposure
SPY
AAL
CBB
SPY
f
SPY^WT/SPY^WT
SPY^WT/SPY^K665A
kDa
25
20
Long exposure
Short exposure
DELLA^1-205
AAL
CBB
100
75
SPY

Supplementary Fig. 6. The *Arabidopsis* SPY dimer. a, Cartoon representative of the dimer interface in *Arabidopsis* SPY. Interface 1 (and interface 1') is shown in red square and interface 2 is shown in blue square. **b,** Details of the dimer interactions are shown. The hydrogen bonds are shown in red dotted line. The hydrophobic interactions are shown in black dotted line. **c-d,** SPY variants (**c**), fucosylated SPY(**c**) and SPY in presence of protein substrate DELLA (**d**) remain dimers in solution as identified by gel filtration assay. Gel filtration profiles were color-coded (Upper panel). SDS-PAGE gels of peak fractions are stained by Coomassie blue (Lower panel). The molecular weights were labeled based on a gel filtration standard (BIO-RAD, catalog #151-1901). Experiments were independently repeated three times with similar results. Uncropped images of Western blotting membranes and gels are available as source data. **e-f**, self-fucosylation (**e**) and fucosylation of DELLA (**f**) by SPY^{WT}/SPY^{WT} homodimer and SPY^{WT}/SPY^{K665A} heterodimer. Experiments were independently repeated three times with similar results. Uncropped images of Western blotting membranes and gels are available as source data.

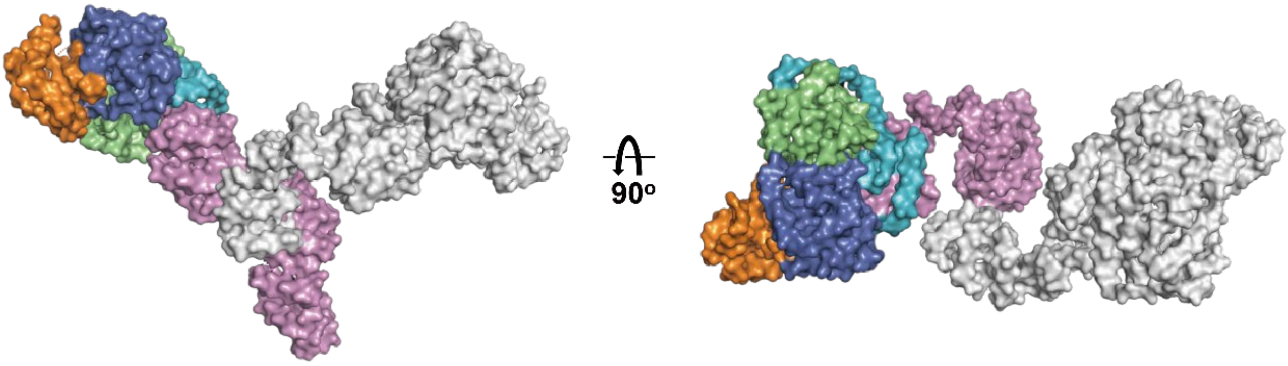

Supplementary Fig. 7. The Human OGT dimer. Two views of the human OGT dimer (PDB code 7NTF) are shown in surface presentation. One protomer is colored the same as in Supplementary Fig. 4A, and the other protomer is colored in gray.

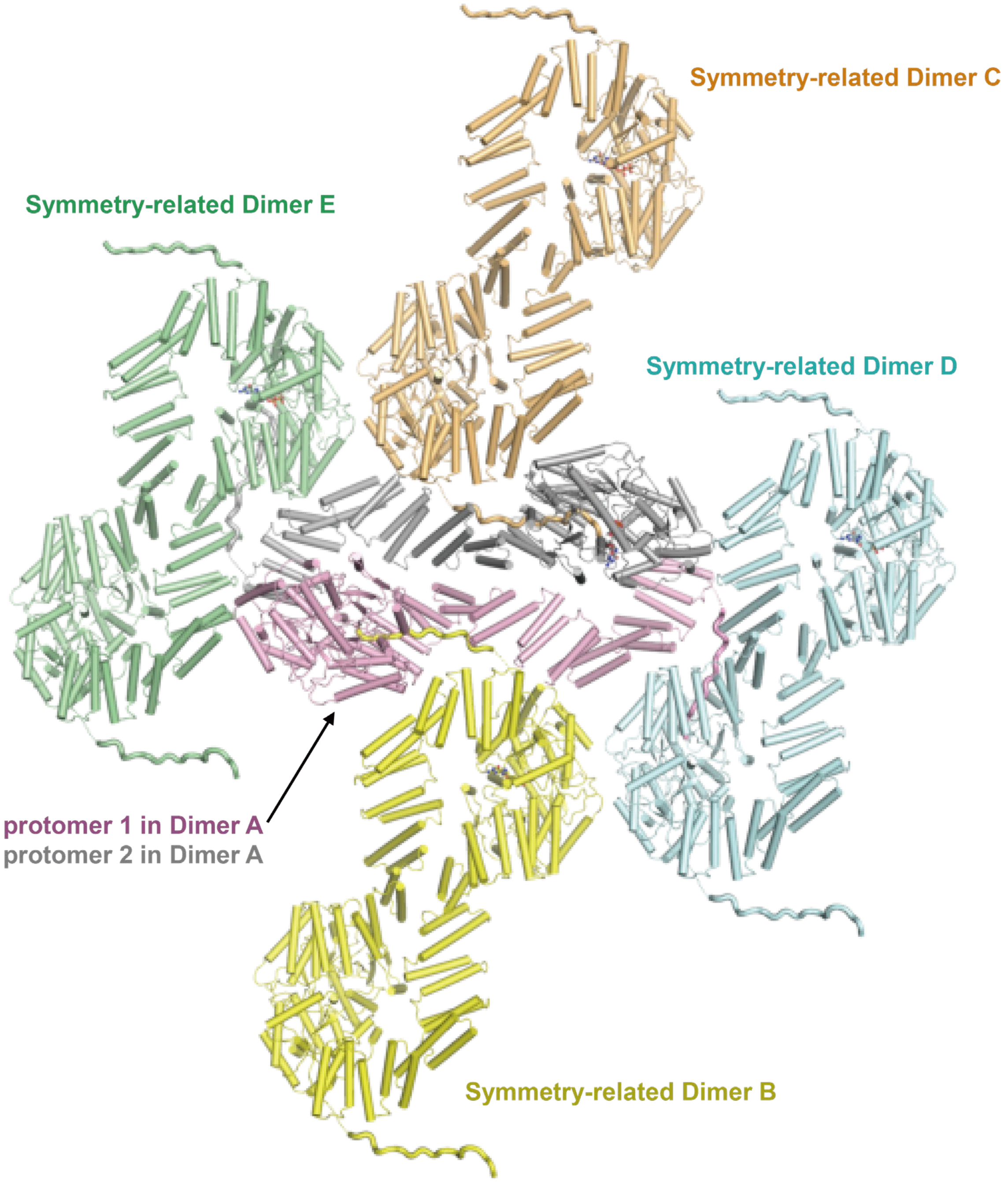

Supplementary Fig. 8. Overview of the SPY dimer to dimer contact in the crystal lattice. Protomers 1 and 2 in SPY dimer A are colored in pink and gray, respectively. Four symmetry-related SPY dimers are colored in yellow, orange, pale cyan and pale green, respectively.

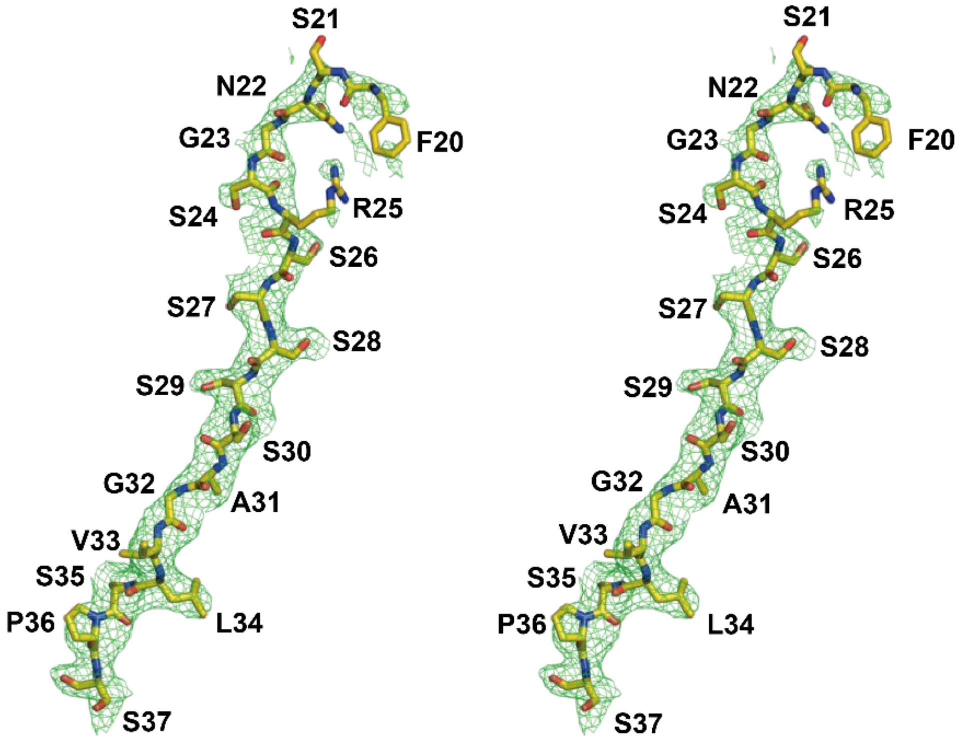

Supplementary Fig. 9. Stereo representation of the electron density map of the N-terminal loop in *Arabidopsis* SPY. The *Fo-Fc* electron density (2.0 σ level) is shown in green.

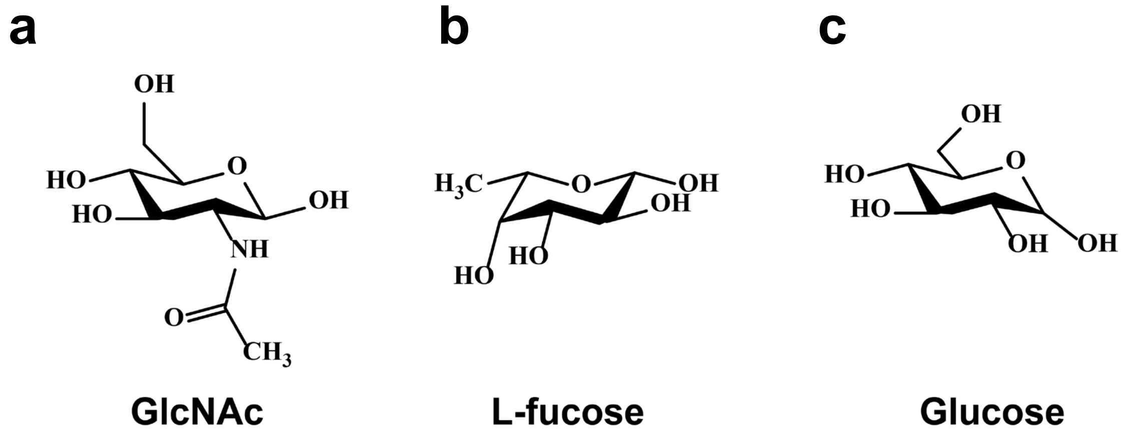

Supplementary Fig. 10. Structures of different hexoses. a, GlcNAc. **b,** L-Fucose. **c,** Glucose.

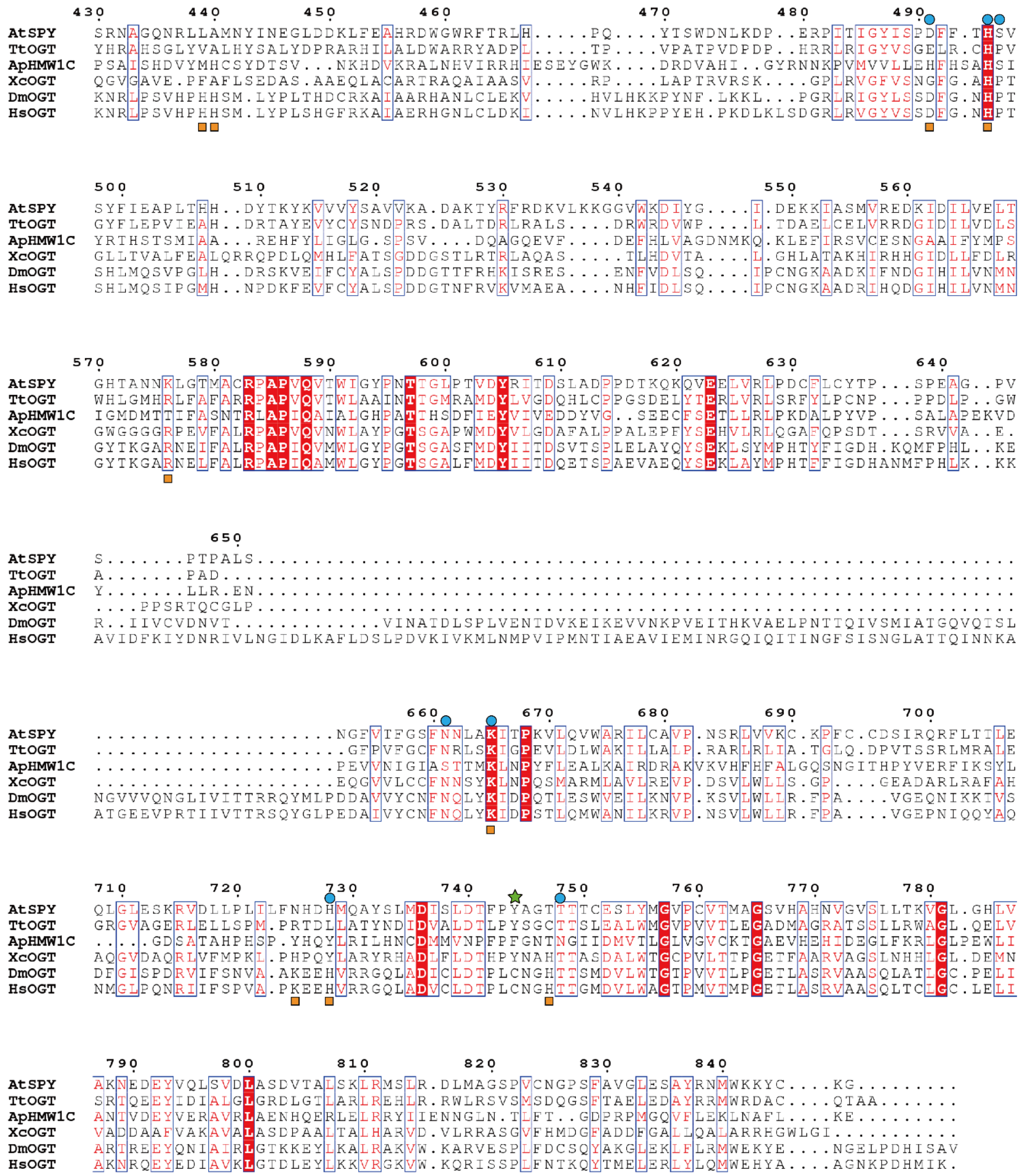

Supplementary Fig. 11. Structure-based sequence alignment of the connector regions and catalytic domains in *Arabidopsis* SPY (AtSPY), *Thermobaculum terrenum* OGT[3] (TtOGT, PDB code 5DJS), *Xanthomonas campestris* OGT[4] (XcOGT, PDB code 2JLB), *Actinobacillus pleuropneumoniae* HMW1C protein[5] (ApHMW1C, PDB code

3Q3H), ***Drososphila melanogaster*** **OGT[6] (DmOGT, PDB code 5A01) and human OGT[7] (HsOGT, PDB code 4N3C).** The green star indicates Y744 in AtSPY. The blue dots and orange squares indicate residues important for enzyme activity in AtSPY and HsOGT, respectively.

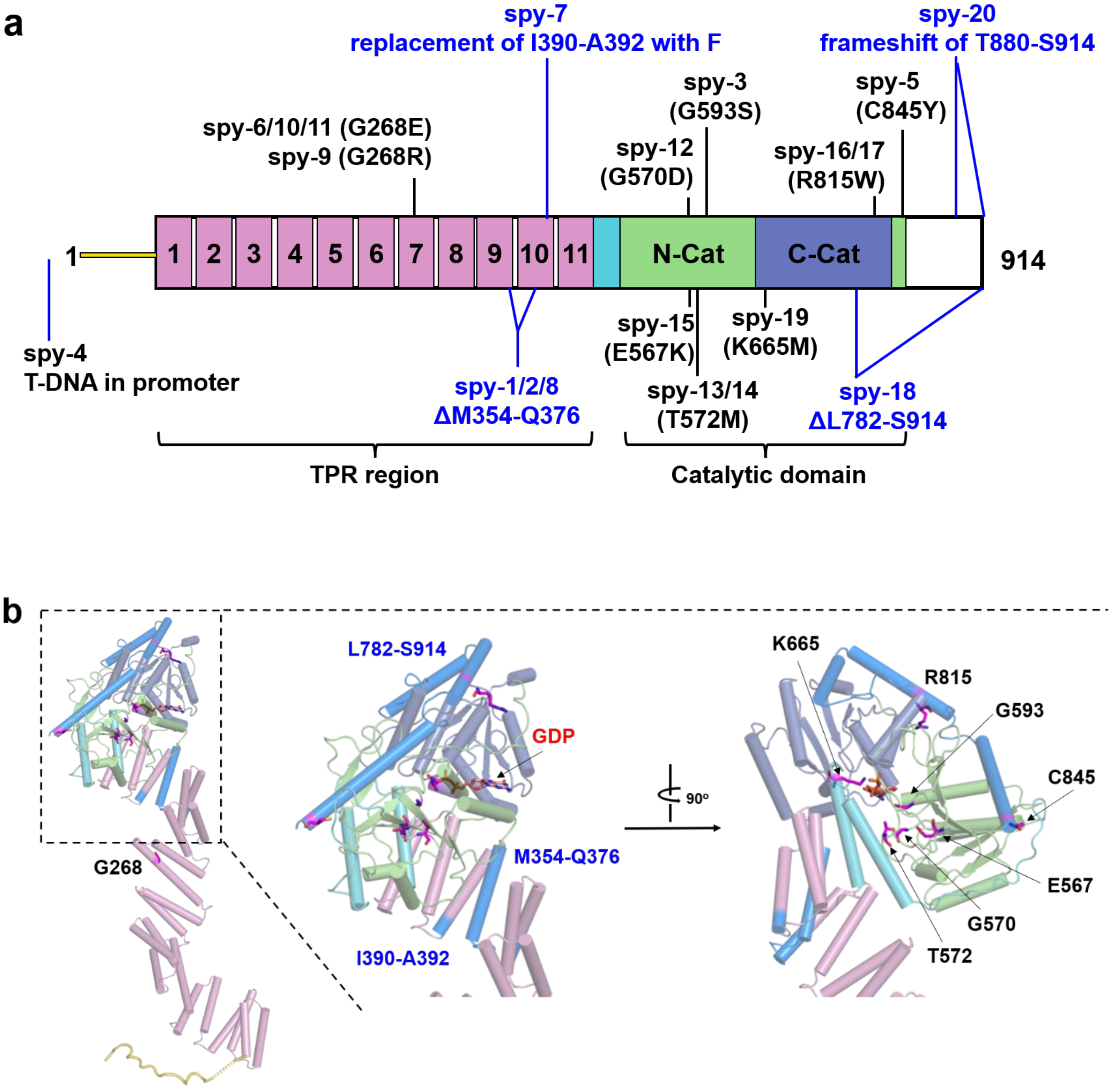

Supplementary Fig. 12. Previously reported SPY mutations. a, Schematic representation of SPY with the nature and location of twenty mutations[8-10] indicated. **b**, The mutations are highlighted in the SPY structure. The color coding for SPY is the same as in Fig. 1c. The point mutations are shown in stick and colored in magenta. The deletion or frameshift region are colored in marine.

Supplementary References

1 Meek, R. W. *et al.* Cryo-EM structure provides insights into the dimer arrangement of the O-linked β-N-acetylglucosamine transferase OGT. *Nature Communications* **12**, 6508, doi:10.1038/s41467-021-26796-6 (2021).

2 Schimpl, M. *et al.* O-GlcNAc transferase invokes nucleotide sugar pyrophosphate participation in catalysis. *Nat Chem Biol* **8**, 969-974, doi:10.1038/nchembio.1108 (2012).

3 Ostrowski, A., Gundogdu, M., Ferenbach, A. T., Lebedev, A. A. & van Aalten, D. M. Evidence for a Functional O-Linked N-Acetylglucosamine (O-GlcNAc) System in the Thermophilic Bacterium Thermobaculum terrenum. *J Biol Chem* **290**, 30291-30305, doi:10.1074/jbc.M115.689596 (2015).

4 Clarke, A. J. *et al.* Structural insights into mechanism and specificity of O-GlcNAc transferase. *EMBO J* **27**, 2780-2788, doi:https://doi.org/10.1038/emboj.2008.186 (2008).

5 Kawai, F. *et al.* Structural Insights into the Glycosyltransferase Activity of the Actinobacillus pleuropneumoniae HMW1C-like Protein*. *Journal of Biological Chemistry* **286**, 38546-38557, doi:https://doi.org/10.1074/jbc.M111.237602 (2011).

6 Mariappa, D. *et al.* Dual functionality of O-GlcNAc transferase is required for Drosophila development. *Open Biol* **5**, 150234, doi:10.1098/rsob.150234 (2015).

7 Lazarus, M. B. *et al.* HCF-1 is cleaved in the active site of O-GlcNAc transferase. *Science (New York, N.Y.)* **342**, 1235-1239, doi:10.1126/science.1243990 (2013).

8 Zentella, R. *et al.* The Arabidopsis O-fucosyltransferase SPINDLY activates nuclear growth repressor DELLA. *Nat Chem Biol* **13**, 479-485, doi:10.1038/nchembio.2320 (2017).

9 Silverstone, A. L. *et al.* Functional analysis of SPINDLY in gibberellin signaling in Arabidopsis. *Plant Physiol* **143**, 987-1000, doi:10.1104/pp.106.091025 (2007).

10 Jacobsen, S. E., Binkowski, K. A. & Olszewski, N. E. SPINDLY, a tetratricopeptide repeat protein involved in gibberellin signal transduction in Arabidopsis. *Proc Natl Acad Sci U S A* **93**, 9292-9296, doi:10.1073/pnas.93.17.9292 (1996).
