# Supplementary material for: Structural insights into mechanism and specificity of the plant protein O-fucosyltransferase SPINDLY
Source: Nat Commun. 2022 Dec 2;13:7424. doi: 10.1038/s41467-022-35234-0 (PMC9715652; doi:10.1038/s41467-022-35234-0)
Supplement: Supplementary file 3 — Description of Additional Supplementary Files [file 41467_2022_35234_MOESM3_ESM.docx]

File Name: Supplementary Data 1

Description: List of PCR primers
